# Vitamin D Receptor Deficiency Enhances Wnt/β-Catenin Signaling and Tumor Burden in Colon Cancer

**DOI:** 10.1371/journal.pone.0023524

**Published:** 2011-08-15

**Authors:** María Jesús Larriba, Paloma Ordóñez-Morán, Irene Chicote, Génesis Martín-Fernández, Isabel Puig, Alberto Muñoz, Héctor G. Pálmer

**Affiliations:** 1 Instituto de Investigaciones Biomédicas "Alberto Sols", Consejo Superior de Investigaciones Científicas-Universidad Autónoma de Madrid, Madrid, Spain; 2 Vall d'Hebrón Institute of Oncology, Stem Cells and Cancer Laboratory, Barcelona, Spain; Roswell Park Cancer Institute, United States of America

## Abstract

Aberrant activation of the Wnt/β-catenin pathway is critical for the initiation and progression of most colon cancers. This activation provokes the accumulation of nuclear β-catenin and the induction of its target genes. *Apc^min/+^* mice are the most commonly used model for colon cancer. They harbor a mutated *Apc* allele and develop intestinal adenomas and carcinomas during the first months of life. This phenotype is caused by the mutation of the second *Apc* allele and the consequent accumulation of nuclear β-catenin in the affected cells. Here we describe that vitamin D receptor (VDR) is a crucial modulator of nuclear β-catenin levels in colon cancer *in vivo*. By appropriate breeding of *Apc^min/+^* mice and *Vdr^+/−^* mice we have generated animals expressing a mutated *Apc* allele and two, one, or none *Vdr* wild type alleles. Lack of *Vdr* increased the number of colonic Aberrant Crypt Foci (ACF) but not that of adenomas or carcinomas in either small intestine or colon. Importantly, colon ACF and tumors of *Apc^min/+^Vdr^-/-^* mice had increased nuclear β-catenin and the tumors reached a larger size than those of *Apc^min/+^Vdr^+/+^*. Both ACF and carcinomas in *Apc^min/+^Vdr^-/-^* mice showed higher expression of β-catenin/TCF target genes. In line with this, *VDR* knock-down in cultured human colon cancer cells enhanced β-catenin nuclear content and target gene expression. Consistently, *VDR* depletion abrogated the capacity of 1,25(OH)_2_D_3_ to promote the relocation of β-catenin from the nucleus to the plasma membrane and to inhibit β-catenin/TCF target genes. In conclusion, VDR controls the level of nuclear β-catenin in colon cancer cells and can therefore attenuate the impact of oncogenic mutations that activate the Wnt/β-catenin pathway.

## Introduction

Colon cancer is the second most common cause of death from cancer in developed countries [1]. Probably, we know more about the genetic alterations causing colon cancer than for any other solid neoplasia. Mutations in *APC*/adenomatous polyposis coli, *CTNNB1*/β-catenin or *AXIN* genes which activate Wnt/β-catenin pathway are responsible for the initiation of most colon cancers [2]. The first step of colon tumorigenesis involves the formation of Aberrant Crypt Foci (ACF), which later progress to adenoma [3]. Posterior malignization of adenoma to carcinoma requires the acquisition of new alterations in genes which are related to Wnt/β-catenin signaling or belong to other pathways that act synergistically in the process [2].

*Apc^min/+^* mice harboring a germ line inactivating mutation in one *Apc* allele develop multiple intestinal adenomas and carcinomas after three months of age [4]. This phenotype occurs as a consequence of spontaneous mutation of the remaining *Apc* allele (loss of heterozygosity) and the activation of the Wnt/β-catenin signaling pathway. This mouse model has become the gold standard of intestinal cancer initiation.

Wnt/β-catenin pathway regulates the ability of β-catenin protein to drive the regulation of specific target genes [5], [6]. In brief, in the absence of a Wnt signal or activating mutations, β-catenin is only present bound to E-cadherin in the intercellular *adherens junctions*, as the free protein is rapidly phosphorylated by casein kinase-Iβ (CK-Iα) (Ser45) and glycogen synthase kinase-3 (GSK-3β) (Ser33, Ser37 and Thr41) in a complex formed also by the tumor suppressor proteins APC and Axin. Phosphorylation labels β-catenin for ubiquitination and degradation by the proteasome [7]. In such basal conditions DNA-bound T-cell factor/lymphoid enhancer factor (TCF/LEF) proteins interact with transcriptional corepressors to block target gene expression in the nucleus. Binding of Wnt ligands to their cell surface receptor complexes (Frizzled-LRP) results in the recruitment of cytoplasmic Axin to the plasma membrane, activation of Dishevelled protein and other not well characterized effects that lead to the inhibition of β-catenin phosphorylation. This allows β-catenin to accumulate and enter into the nucleus, where it interacts with TCF/LEF family members and causes the activation of their otherwise repressed target genes [5], [7]. The Wnt/β-catenin pathway is the main driving force behind the proliferation of epithelial cells in the colon and is essential for the maintenance of progenitor compartments [8], [9]. However, the mutations found in colon cancer result in the aberrant activation of the pathway and induce the constitutive expression of its target genes (mainly involved in cell proliferation and dedifferentiation), leading to the formation of benign yet long-lived adenomas. Accumulation of additional genetic and epigenetic alterations fuels tumor progression.

Many epidemiological and experimental studies indicate that vitamin D_3_ (cholecalciferol), its most active metabolite 1α,25-dihydroxyvitamin D_3_ (calcitriol, 1,25(OH)_2_D_3_) and several analogs protect against colon cancer [10]-[12]. We have reported that 1,25(OH)_2_D_3_ inhibits proliferation and promotes epithelial differentiation of human colon cancer cells by inducing the expression of E-cadherin and by antagonizing the Wnt/β-catenin pathway. The latter is the result of two main mechanisms: a) the induction of direct binding of vitamin D receptor (VDR) to β-catenin, which precludes the formation of transcriptionally active β-catenin/TCF complexes, and b) the induction of β-catenin nuclear export and relocalization at plasma membrane *adherens junctions* as a consequence of E-cadherin upregulation [13]. Additionally, 1,25(OH)_2_D_3_ increases the expression of DICKKOPF-1 (DKK1), a secreted protein that inhibits Wnt signaling from its plasma membrane receptors [14]. We have also described that human *VDR* gene is a direct target of SNAIL1 and SNAIL2/SLUG transcription repressors, and that VDR expression in colon cancer patients is reduced at advanced stages of the disease associated to the upregulation of these factors [15], [16]. Accordingly, high SNAIL1/2 expression in cultured colon cancer cells increases β-catenin transcriptional activity by repressing VDR expression and its antagonistic activity on Wnt/β-catenin signaling [16], [17].

β-Catenin has a wide range of pleiotropic effects that cannot probably be explained solely by the modulation of TCF/LEF action. Thus, β-catenin has been recently described to bind several transcription factors of the nuclear receptor superfamily and homeobox proteins [13], [18], [19]. In most cases, β-catenin binding enhances the transcriptional activity of these factors and affects the expression of alternative or additional sets of target genes involved in cell-fate decisions along development, tissue homeostasis, or cancer [19], [20]. Our initial description of the direct interaction of β-catenin with VDR in human colon cancer cells has been confirmed in other cell systems [21]–[24]. β-catenin/VDR interaction involves the activator function-2 (AF-2) transactivation domain of VDR and the C-terminal domain of β-catenin [21]. In mouse skin, β-catenin/VDR controls target genes, epithelial stem cell fate and tumor development [20]. In this system, increased nuclear β-catenin promoted tumor initiation while VDR ligands protect against cancer by reducing the strength of Wnt/β-catenin signaling [20]. Consistent with this, the treatment of *Apc^min/+^* mice with 1,25(OH)_2_D_3_ or analogs reduces tumor load [25] or polyp number and load [26].

It is important to highlight that the level of β-catenin in the nucleus define the strength of the Wnt signal and in consequence the fate or behavior of several types of normal and tumoral cells [5], [27]. In addition to activating mutations of the Wnt/β-catenin pathway components, other genetic alterations like mutations in *K-RAS* [28] or activation of oncogenic pathways like HGF/c-Met signaling [29] enhance nuclear β-catenin accumulation during colon cancer progression. In such scenario, agents able to diminish β-catenin nuclear content and so to attenuate Wnt/β-catenin signal could be potentially used in cancer therapy as long as tumor cells show Wnt pathway addiction.

In this study we evaluate the consequences of *VDR* deficiency on the initiation and development of intestinal cancer driven by the activation of Wnt/β-catenin pathway. The effect of *VDR* absence has been analyzed both *in vivo* and *in vitro*: in tumors generated in *Apc^min/+^Vdr^-/-^* mice and in cultured human colon cancer cells in which *VDR* expression was knocked-down by means of shRNA. In addition, we have studied the effect of VDR reconstitution in VDR-negative human colon cancer cells. Our results establish a direct link between VDR function and nuclear β-catenin levels that is crucial to control the activity of Wnt/β-catenin signaling in colon cancer *in vivo*.

## Results

**Figure 1:**
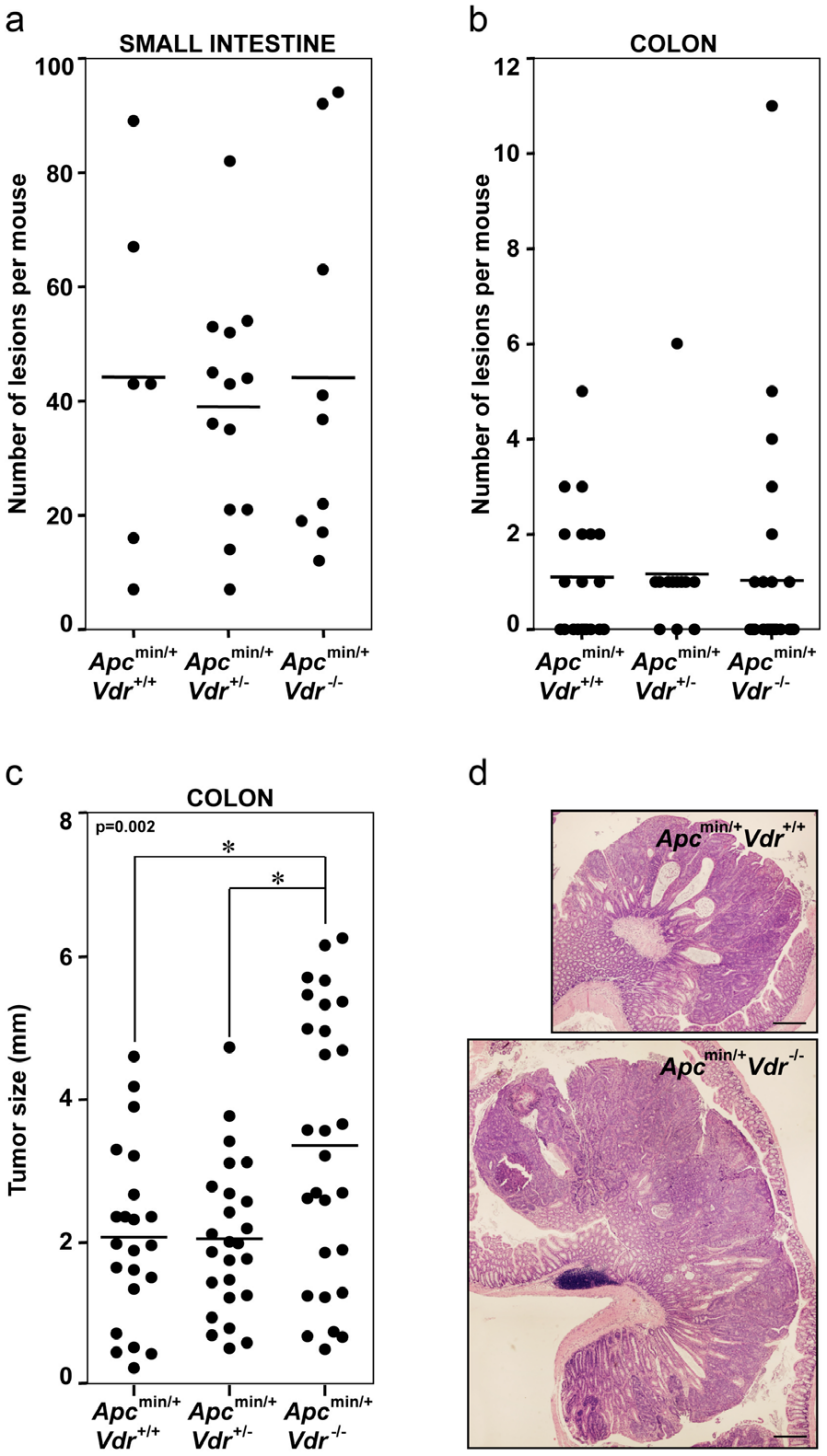
*Vdr* deficiency increases tumor load but not tumor number in *Apc^min/+^* mice. Total number of tumors (adenomas and carcinomas) in the small intestine (a) or in the colon (b) of *Apc^min/+^Vdr^+/+^*, *Apc^min/+^Vdr^+/−^* and *Apc^min/+^Vdr^-/-^* mice. Each dot corresponds to one mouse analyzed and the horizontal line indicates the mean. (c) Size of colon tumors (adenomas and carcinomas) from *Apc^min/+^Vdr^+/+^*, *Apc^min/+^Vdr^+/-^* and *Apc^min/+^Vdr^-/-^* mice. Each dot corresponds to one tumor analyzed and the horizontal line indicates the mean. The p-value for one-way ANOVA analysis is shown. Asterisks indicate significant differences between groups upon Bonferroni multiple comparison post-test. (d) Representative hematoxylin/eosin staining images of colon carcinomas from *Apc^min/+^Vdr^+/+^* and *Apc^min/+^Vdr^-/-^* mice. Scale bar, 300 µm.

**Figure 2:**
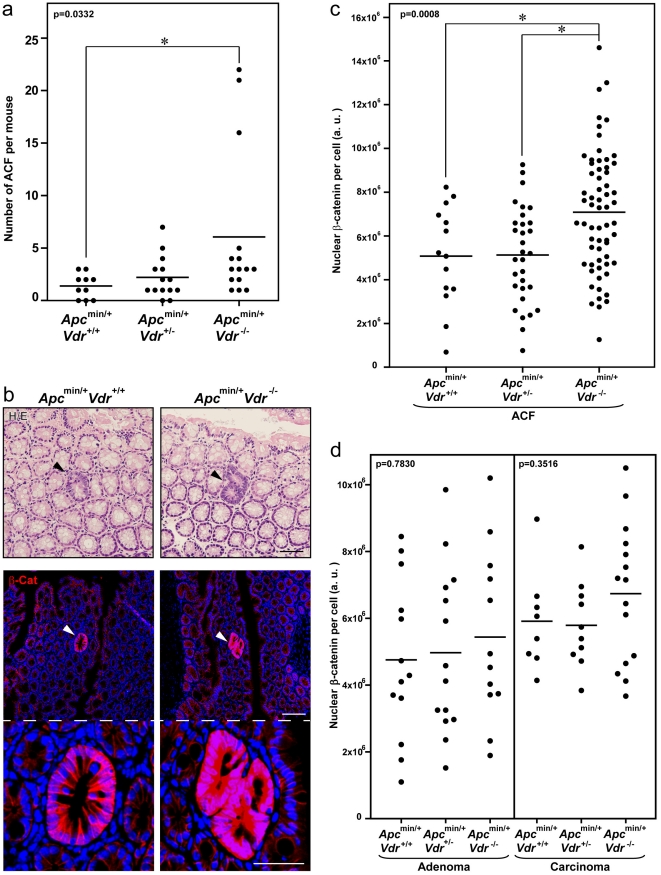
β-Catenin nuclear level is elevated in colonic lesions of *Apc^min/+^Vdr^-/-^* mice. (a) Total number of colonic ACF in *Apc^min/+^Vdr^+/+^*, *Apc^min/+^Vdr^+/−^* and *Apc^min/+^Vdr^-/-^* mice. Each dot corresponds to one mouse analyzed and the horizontal line indicates the mean. (b) Upper panels, representative hematoxylin/eosin staining images of colonic ACF in *Apc^min/+^Vdr^+/+^* and *Apc^min/+^Vdr^-/-^* mice. Arrowheads indicate the ACF. Scale bar, 50 µm. Middle panels, representative immunofluorescence images showing β-catenin expression and localization in colonic ACF from *Apc^min/+^Vdr^+/+^* and *Apc^min/+^Vdr^-/-^* mice. Arrowheads indicate the ACF. Scale bar, 100 µm. Lower panels are a magnification of middle panels. Scale bar, 50 µm. (c) Quantification of β-catenin nuclear level in colonic ACF from *Apc^min/+^Vdr^+/+^*, *Apc^min/+^Vdr^+/-^* and *Apc^min/+^Vdr^-/-^* mice. Each dot corresponds to one lesion analyzed and the horizontal line indicates the mean. (d) Quantification of β-catenin nuclear level in colonic adenomas and carcinomas from *Apc^min/+^Vdr^+/+^*, *Apc^min/+^Vdr^+/-^* and *Apc^min/+^Vdr^-/-^* mice. Each dot corresponds to one lesion analyzed and the horizontal line indicates the mean. (a, c, d) The p-values for one-way ANOVA analysis are shown. Asterisks indicate significant differences between groups upon Bonferroni multiple comparison post-test.

Using available *Apc^min/+^* and *Vdr^+/−^* mice we generated by appropriate crossings *Apc^min/+^Vdr^+/+^, Apc^min/+^Vdr^+/−^* and *Apc^min/+^Vdr^-/-^* animals. Histological analysis at five months of age revealed that *Vdr* deficiency did not affect the total number of tumors in *Apc^min/+^* mice, either in the small intestine or in the colon (Figure 1a, 1b). In contrast, colon tumors in *Apc^min/+^Vdr^-/-^* mice were significantly larger than in *Apc^min/+^Vdr^+/+^* or *Apc^min/+^Vdr^+/−^* mice (Figure 1c). Although different in size, colon adenomas and carcinomas were histopathologically equivalent in all mice independently of their Vdr status (Figure 1d). In addition, the number of colonic ACF was higher in *Apc^min/+^Vdr^-/-^* mice than in *Apc^min/+^Vdr^+/+^* or *Apc^min/+^Vdr^+/-^* mice (Figure 2a).

Next we evaluated the status of Wnt pathway activation in colon lesions by analyzing β-catenin expression. Although ACF in *Apc^min/+^Vdr^+/+^*, *Apc^min/+^Vdr^+/-^* and *Apc^min/+^Vdr^-/-^* mice were indistinguishable by hematoxylin/eosin (H/E) staining, we observed a net increase in nuclear and cytosolic β-catenin levels in the complete absence of *Vdr* (Figure 2b). Whereas β-catenin staining was high and quite homogeneous among cells in ACF (Figure 2b), it was very heterogeneous in carcinomas of the three types of animals (Figure S1a). This phenomenon was also observed in human primary colon carcinomas (Figure S1b), in which the level of nuclear β-catenin is heterogeneous and probably defines the tumorigenic potential of different populations of cancer cells present in the tumor [29]. This heterogeneity may explain why a statistically significant increase in nuclear β-catenin level was found in ACF of *Apc^min/+^Vdr^-/-^* as compared to *Apc^min/+^Vdr^+/+^* or *Apc^min/+^Vdr^+/−^* mice (Figure 2c), but only a minor trend was observed in adenomas and carcinomas (Figure 2d).

**Figure 3:**
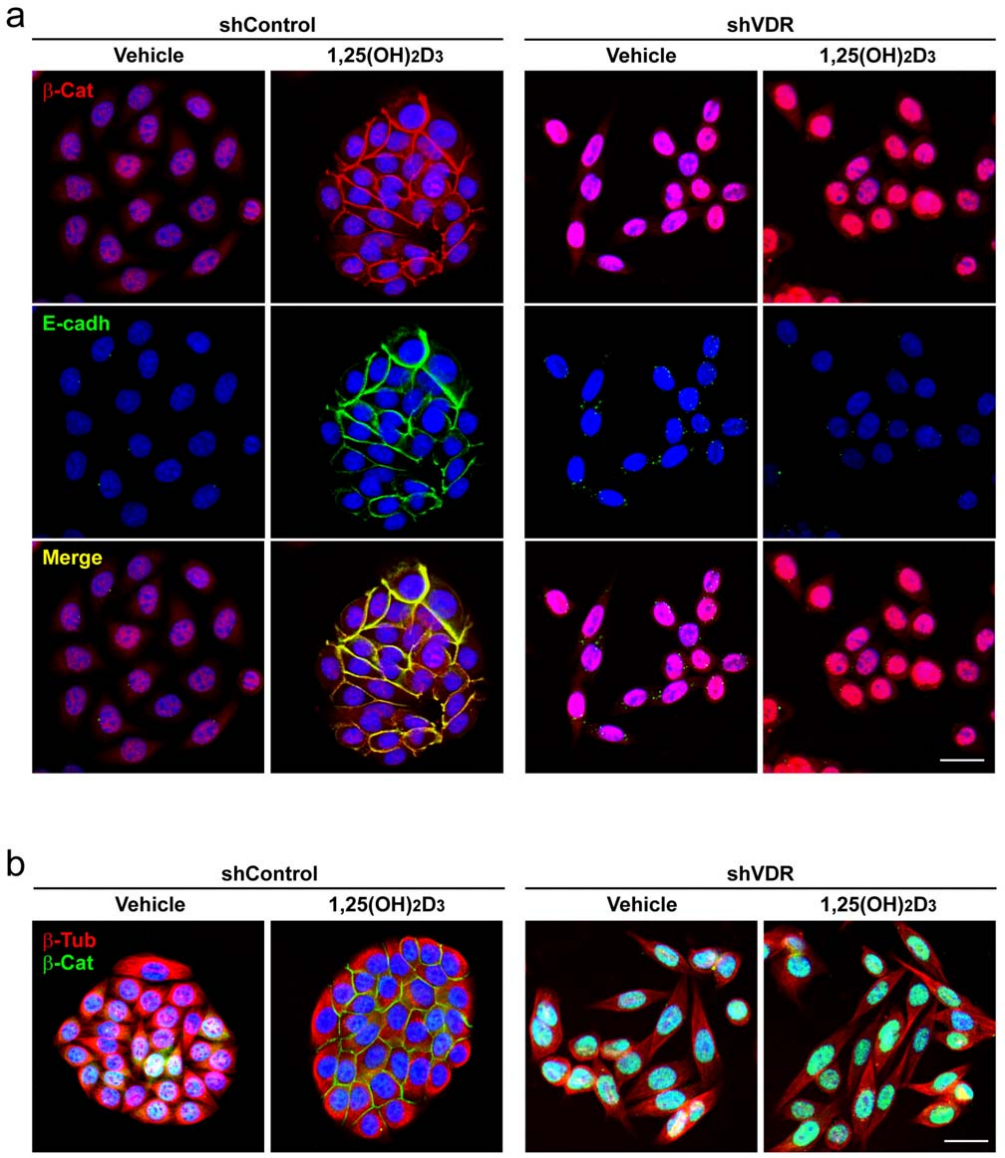
*VDR* knock-down in SW480-ADH human colon cancer cells increases β-catenin nuclear level. Representative immunofluorescence images showing β-catenin and E-cadherin (a) or β-catenin and β-tubulin (b) expression and localization in SW480-ADH cells infected with lentiviruses expressing shVDR or shControl and treated with 1,25(OH)_2_D_3_ or vehicle for 72 h. Scale bar, 20 µm.

To demonstrate a causal link between *VDR* loss and the increase in nuclear β-catenin, we studied SW480-ADH human colon cancer cells in which *VDR* was knocked-down by means of shRNA. These cells harbor most of genetic abnormalities that characterize advanced colon cancers such as mutation of *APC* and *TP53* tumor suppressor genes, activation of *K-RAS* oncogene and *c-MYC* amplification [13]. In agreement with the *in vivo* results, a strong increase in nuclear β-catenin content was found in shVDR SW480-ADH cells as compared to shControl cells (Figure 3a, 3b). In addition, *VDR* knock-down abrogated the capacity of 1,25(OH)_2_D_3_ to induce E-cadherin expression and β-catenin relocation from the nucleus to the plasma membrane (Figure 3a). Also, *VDR* knock-down prevented the reorganization of β-tubulin cytoskeleton and the change in cell morphology induced by 1,25(OH)_2_D_3_ that leads to the formation of compact epithelioid islands (Figure 3b).

**Figure 4:**
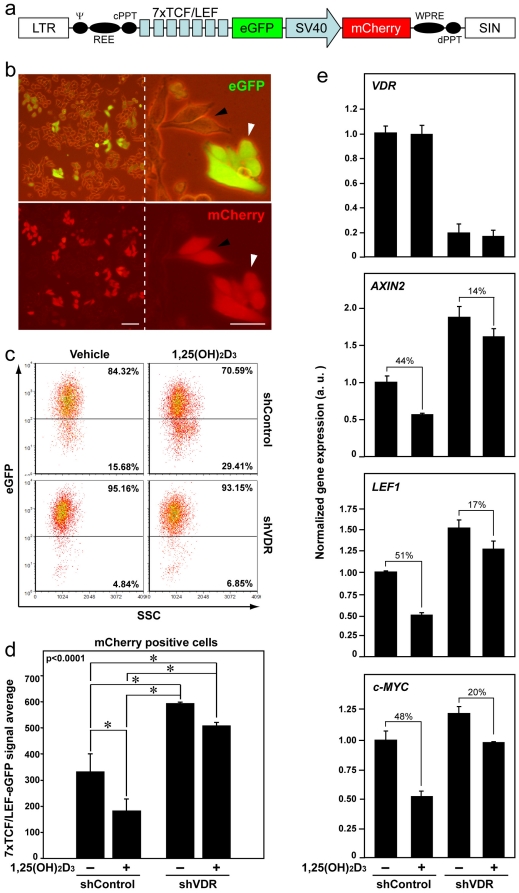
*VDR* knock-down in SW480-ADH cells enhances β-catenin/TCF transcriptional activity and reduces the inhibitory effect of 1,25(OH)_2_D_3_ on Wnt/β-catenin pathway. (a) Diagram of the 7xTCF/LEF-eGFP-SV40-mCherry lentiviral construct used to infect shControl and shVDR SW480-ADH cells. (b) Representative phase-contrast and fluorescence images showing the expression of eGFP and mCherry in SW480-ADH cells infected with the 7xTCF/LEF-eGFP-SV40-mCherry vector. Arrowheads indicate similarly infected cells that have variable expression of eGFP. Scale bars, 20 µm. (c) Flow cytometry analysis of eGFP expression in shControl and shVDR SW480-ADH cells infected (mCherry positive) with the 7xTCF/LEF-eGFP-SV40-mCherry vector and treated with 1,25(OH)_2_D_3_ or vehicle for 96 h. Percentage of cells in each gate is indicated. (d) Quantification of the flow cytometry data showing the average expression of eGFP in the infected mCherry-positive cells. Data correspond to three independent experiments. The p-value for one-way ANOVA analysis is shown. Asterisks indicate significant differences between groups upon Bonferroni multiple comparison post-test. (e) Analysis by qRT-PCR of *VDR*, *AXIN2*, *LEF1* and *c-MYC* mRNA expression in shControl and shVDR SW480-ADH cells treated with 1,25(OH)_2_D_3_ or vehicle for 48 h. The geometric average of the expression of SDHA and TBP housekeeping genes was used for RNA expression normalization as indicated in Materials and Methods. Numbers indicate the percentage of inhibition by 1,25(OH)2D3 treatment in each cell type. Data correspond to three independent experiments.

We wished to analyze whether the increase in the level of nuclear β-catenin translated into a stronger Wnt/β-catenin signaling. To this end, we studied β-catenin/TCF-dependent transcription in colon cancer cells. shControl and shVDR SW480-ADH cells were infected with lentiviruses encoding the reporter eGFP protein under the control of seven copies of a consensus TCF/LEF binding site (Figure 4a) [30]. The presence in the lentiviral construct of the red fluorescent protein mCherry controlled by a constitutive promoter permitted to identify infected cells under a fluorescent microscope and by flow cytometry. Similarly to β-catenin staining in colon tumors, we found certain heterogeneity in β-catenin/TCF activity among the cell culture: variable eGFP expression in equally infected cells (Figure 4b). The analysis of the whole cell population by flow cytometry showed that the percentage of high-eGFP cells was superior in shVDR than in shControl cell cultures (Figure 4c). In addition, shVDR cells had a significantly higher eGFP signal average than shControl cells (Figure 4d). We also found that the decrease in eGFP accumulation caused by 1,25(OH)_2_D_3_ in shControl cells (reduction of the percentage of high-eGFP cells and of eGFP signal average) was attenuated in shVDR cells (Figure 4c, 4d).

Importantly, the elevation of nuclear β-catenin levels was reflected in an increased expression of several β-catenin/TCF target genes: qRT-PCR analysis revealed higher levels of *AXIN2*, *LEF1* and *c-MYC* mRNA in shVDR than in shControl cells (Figure 4e). Additionally, the inhibition of the expression of β-catenin/TCF target genes by 1,25(OH)_2_D_3_ was reduced in shVDR cells (Figure 4e). The reduction of VDR expression by shRNA was also analyzed by qRT-PCR (Figure 4e) and translated into undetectable protein levels ([31] and data not shown). As control we studied the expression of *CDH1*/E-cadherin and *CYP24A1* in these cells, two 1,25(OH)_2_D_3_ target genes. The induction of both genes was drastically inhibited in shVDR cells (Figure S2a).

Next, we extended our studies to other human colon cancer cell lines. *VDR* knock-down in HCT116 and HT29 cells also promoted an increase in nuclear β-catenin levels (Figure S2b) and the activation of TCF/LEF-dependent transcription (Figure S2c), leading to a higher expression of the β-catenin target genes *AXIN2* and *DKK1* (Figure S2d). Such gene regulation was accompanied by a general loss of epithelial organization (Figure S2b). As expected, the reduction of *VDR* expression blocked the capacity of 1,25(OH)_2_D_3_ to induce its target gene *CYP24A1* (Figure S2d). However, and contrarily to data from SW480-ADH cells, 1,25(OH)_2_D_3_ had no effect on β-catenin location or transcriptional activity in HCT116 and HT29 cells (Figure S2d and data not shown). The reason may be that these cells express high level of E-cadherin and thus β-catenin is located at the plasma membrane in the absence of 1,25(OH)_2_D_3_.

Transient expression of exogenous wild type VDR in VDR-negative human SW620 metastatic colon cancer cells reduced their high nuclear β-catenin levels (Figure S3). By contrast, VDR- ΔAF2 and VDR-L417S mutants that are unable to bind β-catenin and recruit classical coactivators [21] did not change the nuclear content of β-catenin (Figure S3). Likewise, the expression of a VDR mutant unable to bind classical coactivators but capable to bind β-catenin (VDR-E420Q) [21] had no effect on β-catenin nuclear content in SW620 cells (Figure S3).

The observed increase in nuclear β-catenin content by *VDR* knock-down is not a consequence of the reduced activity of the kinases CK-Iα or GSK-3β since the level of β-catenin phosphorylation at Ser45 or Ser33/Ser37/Thr41 was unaltered in shVDR SW480-ADH cells (Figure S4a). As β-catenin phosphorylation at Ser552 and Ser675 by protein kinase A and/or AKT has been proposed to promote β-catenin nuclear location and/or transcriptional activity [32]-[34], we also analyzed the possibility that these kinases were involved in the effect of VDR knock-down on β-catenin. We found that β-catenin phosphorylation at Ser552 and Ser675 was reduced in shVDR SW480-ADH cells (Figure S4).

**Figure 5:**
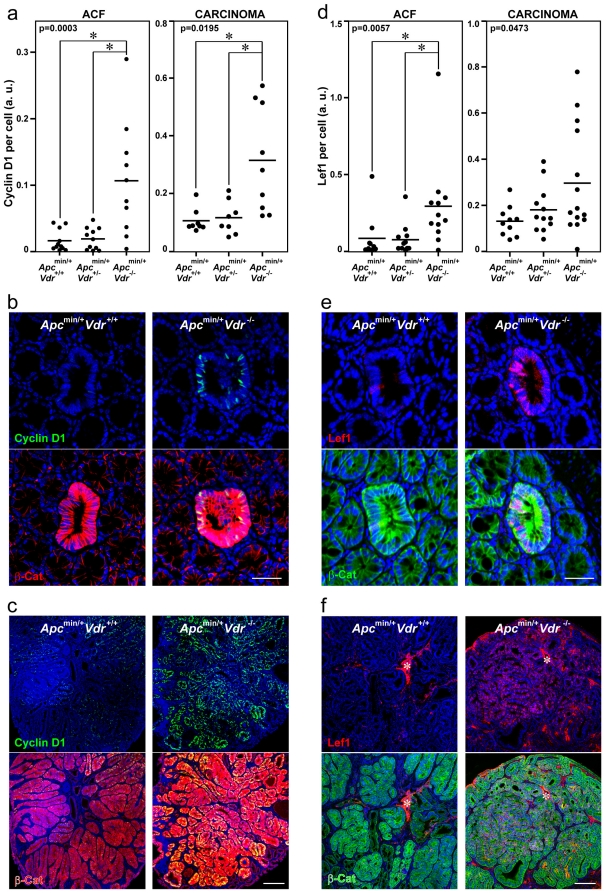
Colonic ACF and carcinomas in *Apc^min/+^Vdr^-/-^* mice have elevated expression of β-catenin/TCF target genes *Ccnd1*/Cyclin D1 and *Lef1*. Quantification of Cyclin D1 (a) and Lef1 (d) protein expression in colonic lesions from *Apc^min/+^Vdr^+/+^*, *Apc^min/+^Vdr^+/-^* and *Apc^min/+^Vdr^-/-^* mice. Each dot corresponds to one lesion analyzed and the horizontal line indicates the mean. The p-values for one-way ANOVA analysis are shown. Asterisks indicate significant differences between groups upon Bonferroni multiple comparison post-test. Representative immunofluorescence images showing β-catenin and Cyclin D1 (b, c) or β-catenin and Lef1 (e, f) expression and localization in colon ACF (b, e) and carcinomas (c, f) from *Apc^min/+^Vdr^+/+^* and *Apc^min/+^Vdr^-/-^* mice. Scale bars, 50 µm (b, e) and 200 µm (c, f). Asterisks indicate an unspecific staining.

Next, we aimed to confirm the effect of VDR deficiency on β-catenin target genes *in vivo*. To this end, we analyzed by immunofluorescence and quantified the level of expression of two β-catenin/TCF target genes (*Ccnd1*/Cyclin D1 and *Lef1*) in colon ACF and carcinomas of *Apc^min/+^Vdr^+/+^*, *Apc^min/+^Vdr^+/-^* and *Apc^min/+^Vdr^-/-^* mice. Accordingly with the data from cultured cells, a significant increased expression of both gene products was found in the lesions of *Apc^min/+^Vdr^-/-^* mice (Figure 5).

**Figure 6:**
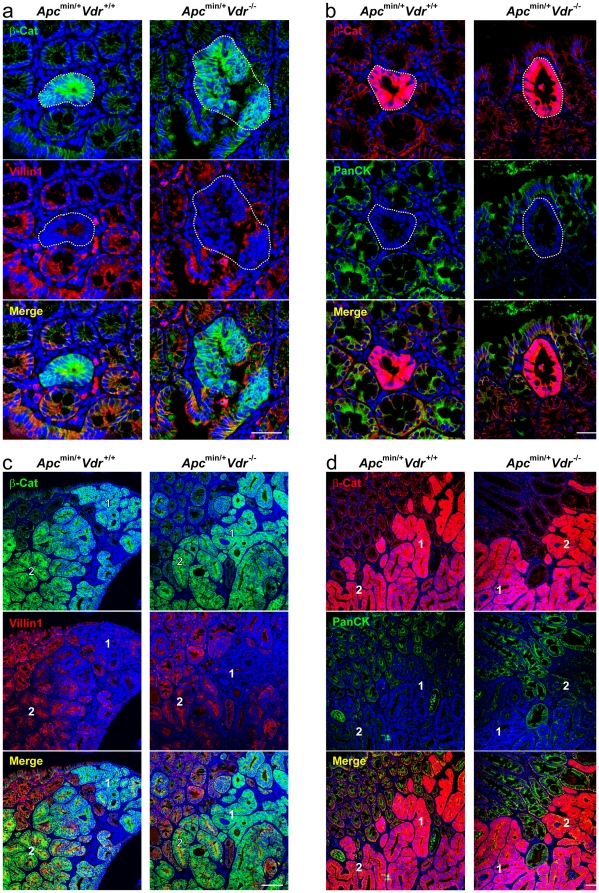
*Vdr* depletion does not affect the differentiation status of colon ACF and carcinomas in *Apc^min/+^* mice. Representative immunofluorescence images showing β-catenin and villin1 (a, c) or β-catenin and pan-cytokeratin (b, d) expression and localization in colon ACF (a, b) and carcinomas (c, d) from *Apc^min/+^Vdr^+/+^* and *Apc^min/+^Vdr^-/-^* mice. Dotted-lines delimit ACF. Numbers indicate regions with different pattern of expression of the analyzed proteins within the same tumor: 1) high β-catenin, low villin1/cytokeratins; 2) low β-catenin, high villin1/cytokeratins. Scale bars, 50 µm (a, b) and 200 µm (c, d).

Finally, we analyzed the degree of differentiation of ACF and carcinomas present in the colon of *Apc^min/+^Vdr^+/+^*, *Apc^min/+^Vdr^+/−^* and *Apc^min/+^Vdr^-/-^* mice. Antibodies against cytokeratins (pan-cytokeratin) and villin1 as markers of epithelial differentiation were used (Figure S5). ACF expressed very low levels of these proteins as expected from the progenitor phenotype imposed by high Wnt/β-catenin signaling (Figure 6a, 6b). In carcinomas the expression of both markers was heterogeneous (Figure 6c, 6d), which agrees with the heterogeneous expression of β-catenin previously described (Figure S1a). No substantial differences in the expression of cytokeratins and villin1 were found between lesions of *Apc^min/+^Vdr^+/+^*, *Apc^min/+^Vdr^+/−^* and *Apc^min/+^Vdr^-/-^* mice, suggesting that these two differentiation markers are lost early during colon tumorigenesis, probably as a consequence of the initial activation of the Wnt/β-catenin pathway.

## Discussion

The level of nuclear β-catenin defines the strength of the Wnt/β-catenin signaling and in consequence the fate and phenotype of many types of normal and cancer cells. Aberrant activation of Wnt/β-catenin signaling due to alterations in components of the pathway is responsible for the initiation of most human colon cancers, which highlights the importance of controlling nuclear β-catenin accumulation [28], [29], [35]. Although the initiation of colon tumorigenesis is considered clonal [36], colon carcinomas show largely heterogeneous nuclear β-catenin expression. This is known as the β-catenin paradox and has led to search for alternative pathways modulating β-catenin location and activity. Among them, *K-RAS* mutation and myofibroblasts-derived HGF have been recently proposed to increase β-catenin nuclear content [28], [29].

Very little is known about mechanisms to diminish the level of β-catenin within the nucleus. We have described that 1,25(OH)_2_D_3_ and several less calcemic analogs interfere the Wnt/β-catenin pathway in a series of human colon cancer cell lines in several modes: they increase the binding of VDR to β-catenin hampering the formation of β-catenin/TCF complexes, induce the expression of the Wnt inhibitor DKK1, and promote the relocation of β-catenin from the nucleus towards the plasma membrane where it binds E-cadherin at *adherens junctions* [13], [14]. Putative mechanisms for this latter effect include the induction of β-catenin nuclear export or the sequester of newly synthesized and/or cytosolic β-catenin protein by E-cadherin, whose expression is strongly induced by 1,25(OH)_2_D_3_. Another mechanism of Wnt/β-catenin inhibition in colon cancer has been proposed by Kaler and cols.: 1,25(OH)_2_D_3_ decreases the synthesis and secretion by THP-1 macrophages of interleukin-1β, a cytokine that activates the Wnt/β-catenin pathway in colon cancer cells through the blockade of β-catenin phosphorylation by GSK-3β [37]. However, the function of all these cell-autonomous and non-cell-autonomous mechanisms *in vivo* remained unknown.

In this study we examined whether *VDR* deficiency alters β-catenin nuclear content and Wnt/β-catenin pathway in the most commonly used animal model for colon cancer, the *Apc^min/+^* mice. Our results show that *Vdr* deficiency in *Apc^min/+^* mice increases nuclear β-catenin levels and expression of Wnt/β-catenin target genes and, in line with these effects, enhances total colon tumor load. Consistently, knocking-down *VDR* by shRNA in human colon cancer cells enhances the nuclear content of β-catenin, its transcriptional activity, and the expression of Wnt/β-catenin target genes. Furthermore, transient restoration of wild type VDR expression in VDR-negative human SW620 colon cancer cells decreases nuclear β-catenin level, whereas VDR-ΔAF2, VDR-L417S or VDR-E420Q mutants, unable to bind classical coactivators and activate gene transcription [21], did not. Curiously, among them only VDR-E420Q is capable to bind β-catenin [21] and its re-expression in *Vdr* ^-/-^ mice rescues alopecia but not rickets phenotype [38]. It seems, therefore, that nuclear β-catenin level and activity depend on the capacity of VDR to recruit classical transcriptional coactivators.

Our data show that VDR knock-down in SW480-ADH cells does not affect β-catenin phosphorylation by CK-Iα or GSK-3β, discarding a role of VDR regulating total β-catenin accumulation. Whereas nuclear β-catenin level increases in the absence of VDR, the total cellular amount of β-catenin protein is not altered (Figure S4a). Unexpectedly, we also found that the phosphorylation of β-catenin at Ser552 and Ser675 proposed to increase β-catenin transcriptional activity is reduced in shVDR SW480-ADH cells. However, the putative inhibitory effect that the reduction of these phosphorylations may have on β-catenin transcriptional activity seems to be overpassed by the effect of VDR deficiency increasing β-catenin nuclear translocation. Altogether, these data suggest that VDR does not control β-catenin degradation but most probably favours its redistribution to the cell nucleus.

Our results reveal a novel *in vivo* function of VDR as crucial modulator of Wnt/β-catenin signal strength in colon cancer. The finding that *VDR* deficiency does not change the number of tumors but increases tumor load indicates that VDR does not block the initial mutations that provoke the early activation of the Wnt/β-catenin pathway, but that it preferentially has a long term protective effect on tumor growth by limiting the strength of the Wnt/β-catenin oncogenic signal. Concordant with our results, a very recent report has shown that *Apc^min/+^Vdr^-/-^* mice present larger intestinal tumors than *Apc^min/+^Vdr^+/+^* mice, although the molecular mechanisms behind this phenotype was not investigated [39]. The concordance of the two parallel studies confirms their main findings.

The results of our study are relevant for the clinic, as VDR expression is downregulated in approximately two-thirds of advanced colon tumors associated to the upregulation of *SNAI1* and *SNAI2* genes that code for SNAIL1/SNAIL2 transcriptional repressors [15], [16], [40]. VDR loss may contribute to overactivate the Wnt/β-catenin pathway and so, to accelerate tumor growth and malignization. A few studies have proposed VDR expression as a marker of good prognostic in colon cancer and showed its correlation with high tumor differentiation and absence of node involvement. However, the low number of patients analyzed does not allow definitive conclusions [41]–[43]. Patients with carcinomas at early stages that still express VDR could benefit from therapy with 1,25(OH)_2_D_3_ compounds that would potentially reduce Wnt/β-catenin oncogenic function. However, when VDR expression is lost at advanced stages, tumors will not only be refractory to this type of treatment but will also acquire higher levels of nuclear β-catenin and enhanced Wnt/β-catenin signaling.

## Materials and Methods

### Ethics statement

Animal work within this article was carried out in strict accordance with the recommendations by the European Union (ECC directive 86/609/EEC, November 1986) and all the experiments were approved by the Ethical Committee for Animal Experimentation of the Consejo Superior de Investigaciones Científicas (Approval ID: PN2010-IE81). All efforts were made to minimize animal suffering. The work involving human samples was conducted according to the principles expressed in the Declaration of Helsinki. The study was approved by the Research Ethics Board of Vall d'Hebrón Institute of Oncology (Approval ID: PR(IR)79/2009). All patients provided written informed consent for the collection of samples and subsequent analysis.

### Cell culture and gene transfer

Human SW480-ADH, HT29, HCT116 and SW620 colon cancer cells were grown in DMEM supplemented with 10% FCS and 2 mM L-glutamine (all from Invitrogen, Carlsbad, CA). SW480-ADH cells are a subpopulation derived from SW480 cell line that express substantial VDR levels and are thus responsive to 1,25(OH)_2_D_3_ [13]. All experiments using 1,25(OH)_2_D_3_ were performed in DMEM supplemented with charcoal-treated FCS to remove liposoluble hormones. Cells were treated with 100 nM 1,25(OH)_2_D_3_ or the corresponding vehicle/isopropanol concentration for the indicated times.

To knock-down *VDR* expression, cells were infected with lentiviral particles containing a shRNA targeting *VDR* (Sigma-Aldrich, St Louis, MO) and selected by puromycin treatment as previously described [31]. shControl cells were infected with lentiviruses bearing a non-targeting shRNA that activates the RISC complex and the RNA interference pathway but that contains at least five mismatched nucleotides compared with any human gene (Sigma-Aldrich). To reconstitute VDR expression, SW620 cells were transiently transfected with the expression vector for human VDR wild type or several mutants (VDR-ΔAF2, VDR-L417S and VDR-E420Q, kindly provided by Dr. A. Aranda, Instituto de Investigaciones Biomédicas, Madrid, Spain) or with the empty vector (pSG5) using jetPEI transfection reagent (PolyPlus Transfection, Illkirch, France), and cells were analyzed after 48 h.

For analysis of β-catenin/TCF transcriptional activity, shControl and shVDR SW480-ADH cells were infected with lentiviruses containing the 7xTCF/LEF-eGFP-SV40-mCherry vector [30] and analyzed by flow cytometry. In the case of shControl and shVDR HCT116 and HT29 cells, they were transiently cotransfected using jetPEI with the pRLTK plasmid (Promega, Madison, WI) containing the *Renilla reniformis* luciferase gene (RLuc) under the control of the *thymidine kinase* promoter and with the TOP-Flash or FOP-Flash plasmid containing four multimerised copies of wild type (TOP-Flash) or mutated (FOP-Flash) TCF/LEF-binding sites upstream of a minimal *c-fos* promoter driven the expression of the *Firefly* luciferase gene (Luc) (kindly provided by Dr. H. Clevers, Hubrecht Laboratory, Utrecht, The Netherlands). Luc and RLuc activities were separately measured 48 h after transfection using the Dual Luciferase Kit (Promega). Luc activity was normalized to RLuc and the TOP-Flash/FOP-Flash ratio calculated.

### Mouse colony and tissue isolation

The *Vdr^+/−^* and *Apc^min/+^* mice (kindly provided by Drs. M. B. Demay, Harvard Medical School, Boston, MA and E. Batlle, Institute for Research in Biomedicine, Barcelona, Spain, respectively) were previously described [4], [44]. *Apc^min/+^Vdr^+/+^*, *Apc^min/+^Vdr^+/−^* and *Apc^min/+^Vdr^-/-^* mice were obtained by appropriate crossings. All mice were kept in a virus- and parasite-free barrier facility. They were exposed to a 12 h light and dark cycle and allowed free access to autoclaved water and chow. At five months of age mice were sacrificed. The mice were dissected and the small intestine and colon removed and flushed with PBS. Intestines were then wound into “Swiss” rolls which were subsequently fixed O/N in 10% neutral-buffered formalin solution (Sigma-Aldrich). Then, tissues were embedded in paraffin, sectioned, deparaffinized, hydrated and stained with hematoxylin/eosin for microscopic tumor scoring. The number of tumors was counted and the size (diameter) of each tumor was measured blindly by two independent researchers using a conventional light microscope.

### Immunofluorescence and confocal microscopy

Cells growing on coverslips were rinsed once in PBS, fixed in cold methanol for 1 min and rinsed in PBS. Non-specific sites were blocked by incubation with PBS containing 5% BSA for 1 h at RT. Then, cells were incubated with the primary antibodies (E-cadherin, BD Biosciences, San Jose, CA; β-catenin, BD Biosciences and Abcam, Cambridge, UK; β-tubulin, Sigma-Aldrich; VDR, Millipore, Billerica, MA) diluted 1/100 in PBS O/N at 4°C. After four washes in PBS, cells were incubated with secondary fluorophore-conjugated antibodies (Invitrogen) and Hoechst 33342 (Sigma-Aldrich) for 1 h at RT, washed and mounted in Vectashield (Vector Laboratories, Burlingame, CA). Cell imaging was performed on a Leica TCS SP5 DMI6000 microscope (Leica, Wetzlar, Germany) using argon ion (488 nm), HeNe (543 nm) and violet diode (405 nm) lasers. Images were acquired sequentially by direct register using LAS AF Leica Confocal Software.

For tissue immunofluorescence, formalin-fixed paraffin-embedded sections of mouse small intestine and colon or human colon tumors were prepared and immunolabelled as described elsewhere [27]. Briefly, tissues were sectioned at 4 µm, deparaffined and rehydrated using xylene and a series of graded ethanol. Antigens were retrieved by microwaving the sections immersed in 10 mM citrate buffer (pH 6.0) for 10 min. Then, sections were cooled at RT for 20 min, rinsed in PBS and permeabilized with 0.2% Triton X-100. Non-specific binding was blocked by incubating the sections in PBS 5% BSA for 1 h at RT. Immunofluorescence staining was carried out incubating the sections with the primary antibodies (β-catenin, BD Biosciences and Abcam; Cyclin D1, Abcam; Lef1, Cell Signaling Technology, Danvers, MA; pan-cytokeratin, Santa Cruz Biotechnology, Santa Cruz, CA; villin1, LifeSpan Biosciences, Seattle, WA) diluted 1/100 in PBS O/N at 4°C. After four washes in PBS, cells were incubated with secondary fluorophore-conjugated antibodies and Hoechst 33342 for 1 h at RT, washed and mounted in Vectashield. Cell imaging was performed as described above for cell immunofluorescence.

For the quantification of the fluorescent signal of tissue immunofluorescences we used the MacBiophotonics ImageJ software. In brief, we defined Regions of Interest (ROI) and obtained the integrated density of the ROI for the green and the red channels corresponding to specific antibody staining. In parallel, we measured the Hoechst integrated density of each ROI and divided it by a standard nucleus signal. The standard nucleus signal was calculated as the average integrated density of ten nuclei taken at random. The resulting values corresponded to the number of nuclei and, therefore, to the number of cells present in each ROI. We finally divided the integrated density of the proteins of interest in the ROI by the number of cells present in the same ROI. This value indicates the level of expression of the proteins of interest per cell. To measure nuclear β-catenin levels we previously used MacBiophotonics ImageJ software to generate an image where the positive staining corresponded to coincident Hoechst (blue channel) and β-catenin (red channel) signals. To quantify the level of nuclear β-catenin in SW620 cells ectopically expressing different VDR variants, we adapted the above mentioned method to single cell measurements. Nuclear β-catenin level (red *vs* blue channel) was calculated in cells transfected with empty vector or with wild type or mutant VDR (green channel). One hundred cells expressing exogenous VDRs (green positive) or the empty vector (green negative) were estimated in each condition and values were represented as arbitrary units. All quantifications were performed blindly by two independent researchers.

### Flow cytometry

One million of shControl and shVDR SW480-ADH cells infected with 7xTCF/LEF-eGFP-SV40-mCherry vector were trypsinized and resuspended in DMEM 10% FCS. Then, cells were washed with PBS and resuspended in 1 ml PBS. DNA was stained with DAPI (2 mg/ml; Sigma-Aldrich) in order to exclude dead, apoptotic or clumped cells. Acquisition was performed using Beckman-Coulter Moflo Cytometer (Fullerton, CA) and the data obtained was analyzed with FCS Express Version 3 Software.

### RNA extraction and quantitative RT-PCR

RNA was extracted from cultured cells using RNeasy Mini Kit (Qiagen, Hilden, Germany) and retrotranscribed using the High-Capacity cDNA Archive Kit (Applied Biosystems, Framingham, MA). Target gene RNA expression was measured by quantitative RT-PCR in relation to the geometric average of two reference housekeeping genes, succinate dehydrogenase complex subunit A (*SDHA*) and TATA box binding protein (*TBP*), following the recommendations by Vandesompele et al. [45]. Oligonucleotides for *VDR*, *CDH1* and *SDHA* were previously reported [15], *AXIN2* (sense 5′-AGTCAGCAGAGGGACAGGAA-3′, antisense 5′-GTGGACACCTGCCAGTTTCT-3′), *LEF1* (sense 5′-CGAAGAGGAAGGCGATTTAG-3′, antisense 5′-GTCTGGCCACCTCGTGTC-3′), *c-MYC* (sense 5′-TGGATTTTTTTCGGGTAGTGG-3′, antisense 5′-GTCGTAGTCGAGGTCATAGTTCC-3′) and *TBP* (sense 5′-CGGCTGTTTAACTTCGCTTC-3′, antisense 5′-CACACGCCAAGAAACAGTGA-3′). Primers were designed to recognize regions of the mRNAs codified by different exons to avoid genomic DNA amplification. In addition, control samples in which reverse transcription was performed without reverse transcriptase were always included in the quantitative PCR runs. The level of *CYP24A1* and *DKK1* RNA were measured in relation to that of *18S* rRNA using the comparative C_T_ method and TaqMan probes (Applied Biosystems). The quantitative PCR reaction was performed in an ABI Prism 7900 HT thermal cycler using SYBR or TaqMan Gene Expression Master Mixes (all from Applied Biosystems). Thermal cycling consisted of a denaturing step at 95°C for 10 min and 40 cycles of denaturing at 95°C for 15 s and annealing and elongation at 60°C for 60 s.

### Western blotting

Whole-cell extracts were prepared by washing the monolayers twice in PBS and cell lysis by incubation in RIPA buffer (50 mM Hepes pH 7.4, 150 mM NaCl, 1.5 mM MgCl_2_, 10% glycerol, 4 mM EDTA, 1% Triton X-100, 0.1% SDS, 1% deoxycholate) plus phosphatase- and protease-inhibitor mixture (25 mM β-glycerophosphate, 1 mM Na_3_VO_4_, 10 mM NaF, 1 mM PMSF, 10 µg/ml leupeptin, 10 µg/ml aprotinin) for 15 min on ice followed by centrifugation at 13,000 rpm for 10 min at 4°C. Western blotting of cell lysates was performed by electrophoresis in SDS-PAGE and protein transfer to Immobilon P membranes (Millipore). The membranes were incubated with the appropriate primary and secondary horseradish peroxidase-conjugated antibodies, and the antibody binding was visualized using the ECL detection system (GE Healthcare, Chalfont St Giles, UK). The following primary antibodies were used: mouse monoclonal against E-cadherin, β-catenin (BD Biosciences) and active-β-catenin (anti-ABC) (Millipore); rat monoclonal against VDR (Millipore); and rabbit polyclonal against phospho-β-catenin-Ser552, phospho-β-catenin-Ser45, phospho-β-catenin-Ser675, and phospho-β-catenin-Ser33/Ser37/Thr41 (Cell Signaling). Quantification was done by densitometry using ImageJ software.

### Statistical analysis

Statistical significance was assessed by unpaired *t* test with Welch's correction when two groups of values were compared. For multiple group comparisons we used an unpaired one-way ANOVA analysis with Bonferroni multiple comparison post-test. All calculations were performed using the GraphPad Prism 5 software (San Diego, CA). Differences were considered significant when p<0.05.

## Supporting Information

Figure S1 **β-Catenin expression and localization is heterogeneous in colon carcinomas from** ***Apc^min/+^*** **mice and in human colon tumors.** (a) Representative immunofluorescence image showing β-catenin expression and localization in colon carcinomas from *Apc^min/+^* mice. Numbers indicate regions with different pattern of β-catenin expression and localization: 1) high cytosolic and nuclear; 2) low membrane and cytosolic. Scale bar, 500 µm. (b) Representative immunofluorescence images showing β-catenin expression and localization in human colon tumors. Numbers indicate regions with different pattern of β-catenin expression and localization within the same tumor: upper panel: 1) membrane and cytosolic; 2) very high cytosolic and nuclear; 3) high membrane and cytosolic; lower panel: 1, membrane; 2, high cytosolic and nuclear. Scale bar, 100 µm.
(TIF)

Figure S2 ***VDR*** **knock-down in human HCT116 and HT29 colon cancer cells increases β-catenin nuclear levels and transcriptional activity.** (a) Analysis by qRT-PCR of *CYP24A1* and *CDH1*/E-cadherin mRNA expression in shControl and shVDR SW480-ADH cells treated with 1,25(OH)_2_D_3_ or vehicle for 48 h. Data correspond to three independent experiments. (b) Representative immunofluorescence images showing β-catenin expression and localization in HCT116 and HT29 cells infected with lentiviruses expressing shVDR or shControl. Scale bar, 100 µm. (c) Cells were transfected with the wild type (TOP-Flash) or mutated (FOP-Flash) β-catenin/TCF reporter plasmid and luciferase activity was measured 48 h later. Values were represented as the TOP-Flash/FOP-flash ratio and related to shControl cells. Data correspond to five independent experiments. The p-values for unpaired *t* test with Welch's correction are indicated. (d) Analysis by qRT-PCR of *VDR*, *CYP24A1*, *AXIN2* and *DKK1* mRNA expression in shControl and shVDR HCT116 and HT29 cells treated with 1,25(OH)_2_D_3_ or vehicle for 48 h. Data correspond to three independent experiments. (a, d) The geometric average of the expression of SDHA and TBP housekeeping genes (for CDH1, VDR and AXIN2) or the expression of 18S rRNA (for CYP24A1 and DKK1) was used for RNA expression normalization as indicated in Materials and Methods.
(TIF)

Figure S3 **Exogenous wild type VDR expression reduces nuclear β-catenin levels in SW620 cells.** (a) Representative immunofluorescence images showing β-catenin and VDR expression and localization in SW620 cells transiently transfected with wild type (WT) VDR, or the VDR-ΔAF2, VDR-L417S or VDR-E420Q mutants for 48 h. Scale bar, 50 µm. (b) Quantification of nuclear β-catenin level in SW620 cells transiently transfected with empty vector (pSG5), wild type VDR or the indicated VDR mutants for 48 h. One hundred cells per condition were studied. Each dot corresponds to one cell analyzed and the horizontal line indicates the mean. The p-value for one-way ANOVA analysis is shown. Asterisks indicate significant differences between groups upon Bonferroni multiple comparison post-test.
(TIF)

Figure S4 **Effect of VDR absence on β-catenin phosphorylation by several kinases.** (a) Western blot analysis of β-catenin phosphorylation in residues targeted by GSK-3β (Ser33, Ser37 and Thr41), CK-Iα (Ser45), PKA (Ser552 and Ser675) or AKT (Ser552) in SW480-ADH shControl and shVDR cells treated with 1,25(OH)_2_D_3_ or vehicle for 72 h. Antibodies that specifically recognize each type of phosphorylated β-catenin or the unphosphorylated protein in Ser37 or Thr41 (active-β-catenin) were used. VDR and E-cadherin expression in the same extracts was shown as a control. A representative experiment of three performed is shown. (b) Quantification of the data obtained in three independent experiments performed as in (a). Phosphorylated β-catenin levels were normalized to total β-catenin.
(TIF)

Figure S5 **Pan-cytokeratin and villin1 are differentiation markers in mouse colon tissue.** Representative immunofluorescence images showing β-catenin and pan-cytokeratin (a) or β-catenin and villin1 (b) staining of mouse normal colon tissue. Both pan-cytokeratin and villin1 markers stain the differentiated cells located at the upper part of the normal mouse colon crypts. Scale bar, 50 µm.
(TIF)
